# Flies and Ants from Domestic Kitchens as Sources of Clinically Important Bacteria and Antimicrobial Resistance

**DOI:** 10.3390/microorganisms14091928

**Published:** 2026-09-01

**Authors:** Carolina Magri Ferraz, Valéria Modolo Peterle, Matheus Zorzal Bernardes Rangel, Gabrielly de Moura Paris, Enzo Bernardes Rocha Fávaro, Sarah Bernardes Simões, Lucas Possa Oliveira, Heloisa Cristina Brugnera, Júlia Silva Santana, Gustavo Guimarães Fernandes Viana, Alessandra Figueiredo de Castro Nassar, Vanessa Castro, Ricardo Pinto Schuenck, Juliano Gonçalves Pereira, João Pedro Rueda Furlan, Marita Vedovelli Cardozo, Gabriel Augusto Marques Rossi

**Affiliations:** 1 Department of Veterinary Medicine, University Vila Velha (UVV), Av. Comissário José Dantas de Melo, N.21, Vila Velha 29102-920, ES, Brazil; carolina.ferraz@uvv.br (C.M.F.); valeriapeterle@gmail.com (V.M.P.); matheuszorzalbernardesrangel@gmail.com (M.Z.B.R.); gabriellyparis@icloud.com (G.d.M.P.); enzobernardes@hotmail.com (E.B.R.F.); sarahbsacademico@gmail.com (S.B.S.); lucaspossaoliveira@gmail.com (L.P.O.); 2 Department of Pathology, Reproduction and One Health, Faculty of Agricultural and Veterinary Sciences, São Paulo State University (UNESP), Jaboticabal 14884-900, SP, Brazil; heloisa.brugnera@unesp.br (H.C.B.); marita.vedovelli@unesp.br (M.V.C.); 3 Molecular Biology and Bacterial Virulence Laboratory, Department of Pathology, Health Sciences Center, Federal University of Espírito Santo, Av. Marechal Campos, s/no, Maruípe, Vitória 29043-900, ES, Brazil; jsantana.avlis@gmail.com (J.S.S.); ricardo.schuenck@ufes.br (R.P.S.); 4 Department of Animal Production and Preventive Veterinary Medicine, School of Veterinary Medicine and Animal Science, São Paulo State University (UNESP), Botucatu 18618-681, SP, Brazil; ggf.viana@unesp.br (G.G.F.V.); juliano.pereira@unesp.br (J.G.P.); 5 Instituto Biológico (IB) de São Paulo, São Paulo 04014-002, SP, Brazil; afcnassar@sp.gov.br (A.F.d.C.N.); vanessa.castro@sp.gov.br (V.C.); 6 Department of Pharmaceutical Sciences, Health Sciences Center, Federal University of Paraíba, João Pessoa 58051-900, PB, Brazil; joao.furlan@academico.ufpb.br

**Keywords:** antimicrobial-resistant bacteria, Enterobacterales, *Staphylococcus*, house flies, ants, food hygiene

## Abstract

Foods can be contaminated by a wide range of microorganisms, originating from multiple sources within domestic kitchens. However, the role of flies and ants as carriers of bacteria in these environments remains poorly understood, and data on the antimicrobial susceptibility profiles of insect-associated bacteria remain scarce. Therefore, this study aimed to identify Enterobacterales and *Staphylococcus* spp. recovered from flies and ants collected in domestic kitchens and to characterize the antimicrobial susceptibility profiles of the isolates. Additionally, selected virulence genes were investigated in *Staphylococcus aureus* and *Escherichia coli*, two recognized foodborne pathogens. A total of 240 insects (113 flies and 127 ants) were collected from 58 domestic kitchens. Twenty-six Enterobacterales isolates belonging to nine species were recovered from 17 kitchens, including a single multidrug-resistant (MDR) isolate identified as *Enterobacter hormaechei*. In addition, 45 staphylococcal isolates representing nine species were recovered from 25 kitchens, originating from 36 flies and 9 ants. Among these, 13 isolates exhibited an MDR phenotype, including *S. aureus* and several non-*aureus Staphylococcus* species. None of the *E. coli* isolates carried the *eae* or *stx1/stx2* genes, whereas none of the *S. aureus* isolates harbored the *sea*, *seb*, *sec*, *see*, or *mecA* genes. To the best of our knowledge, this is the first study to report the simultaneous recovery of MDR bacteria from flies and ants collected in domestic kitchens. These findings provide new evidence that household insects may act as carriers of antimicrobial-resistant bacteria, highlighting their potential role in the dissemination of clinically relevant bacterial species, including MDR strains, within domestic environments. Effective insect control and good household food hygiene practices may help reduce the spread of antimicrobial-resistant bacteria and mitigate the risk of difficult-to-treat foodborne infections.

## 1. Introduction

Antimicrobial resistance (AMR) is considered one of the greatest threats to global public health, contributing to increased morbidity, mortality, and healthcare costs in both human and veterinary medicine. Addressing this challenge requires a multidisciplinary and coordinated approach that includes the prudent use of antimicrobials, the implementation of effective public health policies, and continuous surveillance across human, animal, and environmental sectors through a One Health approach [1,2].

Although AMR is relevant across a wide range of microorganisms, antimicrobial-resistant Enterobacterales and *Staphylococcus* species, particularly those resistant to critically important antimicrobial agents, are considered among the priority pathogens for surveillance and control efforts [3]. Among Enterobacterales, species such as *Escherichia coli*, *Klebsiella pneumoniae*, *Enterobacter cloacae* complex, and *Serratia marcescens* are recognized as major opportunistic pathogens associated with a wide range of community- and healthcare-associated infections [4]. Furthermore, *Staphylococcus aureus* is particularly notable due to its prominent role in difficult-to-treat infections [5]. Nevertheless, several coagulase-negative staphylococci (CoNS) have also emerged as important opportunistic pathogens that cause a wide range of infections, especially in healthcare-associated settings [6]. Beyond their roles in tissue infections, *Enterobacteriaceae* and *S. aureus* are recognized as important causes of foodborne diseases associated with the contaminated foods [7,8].

Foods may become contaminated with antimicrobial-resistant bacteria (ARBs) from multiple sources during kitchen handling and preparation. Potential contamination sources include: (i) food-contact surfaces such as sinks, cutting boards, and countertops; (ii) utensils and cleaning materials, including sponges and cloths; (iii) kitchen equipment such as refrigerators [9,10,11,12]; (iv) food handlers [13]; and (v) insects [14,15]. Flies are frequently associated with human-inhabited environments and regularly contact contaminated substrates, including animal waste, decomposing organic matter, and food residues. Their feeding habits, morphology, and mobility facilitate the acquisition and spread of bacteria [16]. Similarly, ants have also been reported as carriers of foodborne pathogens in kitchen environments, highlighting their potential role in the contamination of food and food-contact surfaces. Their small body size allows them to access cracks, crevices, food storage areas, and food-contact surfaces, while their highly mobile and social foraging behavior promotes frequent movement between contaminated sites and food preparation areas [15].

The presence of ARBs in flies has been reported in slaughterhouses and meat-processing facilities [16], hospitals [17], areas surrounding healthcare facilities [18], livestock farms [19], and kitchens [14]. Despite increasing evidence that flies can harbor ARBs, information regarding the occurrence and AMR profiles of bacteria carried by insects in domestic kitchens remains scarce [20]. In addition, reports on ants as carriers of ARBs are still largely restricted to hospital environments [21]. Thus, this study aimed to investigate flies and ants as carriers of ARBs in domestic kitchen environments in Brazil. The combination of MALDI-TOF MS for bacterial identification, phenotypic antimicrobial susceptibility testing, and PCR-based detection of antimicrobial resistance and virulence genes provides a comprehensive approach for the phenotypic and genotypic characterization of ARBs carried by these vectors in domestic environments.

## 2. Materials and Methods

### 2.1. Sampling

The study was conducted during 2024 in 58 domestic kitchens located in the Metropolitan Region of Vitória, state of Espírito Santo, Southeastern Brazil. In each kitchen, a maximum of five flies and/or five ants were collected, depending on the availability of insects at each visit. A total of 240 insects were analyzed in this study, comprising 113 flies and 127 ants. Flies were collected from 41 kitchens, whereas ants were collected from 36 kitchens. Among the 58 domestic kitchens included in the study, both insect groups were simultaneously collected in 19 kitchens, while only one insect group (either flies or ants) was obtained from the remaining 39 kitchens (Supplementary Table S1). These insects were collected directly from kitchen surfaces using sterile flasks. Each flask was carefully placed over the insects and immediately sealed, without the use of any additional instruments for handling or capture. After collection, the insects were transported and stored in a commercial freezer at −18 ± 2 °C for approximately 2 h until the insects’ death and subsequently processed within 24 h [16]. Precise taxonomic identification of the insect species was not performed to avoid unnecessary handling and the potential risk of sample contamination.

### 2.2. Bacterial Isolation and Identification

Individual insects were aseptically transferred to sterile tubes containing 5 mL of Brain Heart Infusion (BHI) broth (Kasvi^®^, São José dos Pinhais, PR, Brazil) and incubated at 35 ± 2 °C for 24 h. Following incubation, the tubes were homogenized using a vortex mixer, and a 100-µL aliquot of the enriched broth was streaked onto Eosin Methylene Blue (EMB) agar (Kasvi^®^, São José dos Pinhais, PR, Brazil) and Mannitol Salt Agar (MSA) (Kasvi^®^, São José dos Pinhais, PR, Brazil). The inoculated plates were incubated at 35 ± 2 °C for 24 h. EMB agar was selected because the initial focus of the study was the recovery of *E. coli*; therefore, only colonies exhibiting a characteristic metallic green sheen were considered presumptive. On MSA, colonies displaying yellow, whitish, grayish, or slightly translucent morphology were regarded as presumptive of staphylococci. Representative colonies with distinct morphologies were selected from each culture plate for identification by using matrix-assisted laser desorption/ionization time-of-flight mass spectrometry (MALDI-TOF MS) on the Microflex LT/SH system (Bruker, Billerica, MA, USA). For genus- and species-level identification, only isolates yielding a MALDI-TOF MS score ≥ 2.0 were included in the study. Isolates with scores below this threshold were excluded because reliable species-level identification was required for antimicrobial susceptibility testing and interpretation. This is particularly important because the applicable clinical breakpoints may vary among species within the same genus, as observed, for example, among *Staphylococcus* spp.; therefore, the antimicrobial susceptibility results cannot be reliably interpreted based solely on genus-level identification (scores lower than 2.0). After identification, all isolates were preserved in BHI broth supplemented with 15% glycerol and stored at −18 ± 2 °C for subsequent analyses.

### 2.3. Antimicrobial Susceptibility Testing

Antimicrobial susceptibility was evaluated by the disk diffusion method. Prior to antimicrobial susceptibility testing (AST), Enterobacterales and *Staphylococcus* spp. isolates were, respectively, cultured on MacConkey agar and MSA (Kasvi^®^, São José dos Pinhais, PR, Brazil) and incubated at 35 ± 2 °C for 18 ± 2 h. Bacterial suspensions were subsequently prepared and standardized to a turbidity equivalent to a 0.5 McFarland standard using a Densimat instrument (bioMérieux, Craponne, France). The standardized inoculum was then uniformly distributed over the surface of Mueller–Hinton agar (Kasvi^®^, São José dos Pinhais, PR, Brazil) using a sterile cotton swab. Finally, antimicrobial disks were placed onto the agar according to the bacterial group under investigation (Enterobacterales or *Staphylococcus* spp.), and the plates were incubated at 37 °C for 24 h until the diameters of the inhibition zones were measured. For disk quality control, the strains *S. aureus* ATCC^®^ 29213 and *Escherichia coli* ATCC^®^ 25922 were used.

AST of Enterobacterales and *Staphylococcus* spp. was performed using panels of up to 15 antimicrobial agents. Enterobacterales: amoxicillin–clavulanic acid (AMC, 30 µg), aztreonam (ATM, 30 µg), imipenem (IPM, 10 µg), meropenem (MPM, 10 µg), ceftazidime (CAZ, 30 µg), ceftriaxone (CRO, 30 µg), cefepime (CPM, 30 µg), streptomycin (STP, 10 µg), gentamicin (GEN, 10 µg), doxycycline (DOX, 30 µg), tetracycline (TET, 10 µg), ciprofloxacin (CIP, 5 µg), trimethoprim–sulfamethoxazole (SXT, 25 µg), chloramphenicol (CHL, 30 µg), and nitrofurantoin (NIT, 300 µg). *Staphylococcus* spp.: penicillin (PEN, 10 units), cefoxitin (CFO, 30 µg), amikacin (AMI, 30 µg), GEN (10 µg), doxycycline (DOX, 30 µg), TET (10 µg), enrofloxacin (ENR, 5 µg), SXT (25 µg), CHL (30 µg), rifampicin (RIF, 5 µg), clindamycin (CLI, 2 µg), azithromycin (AZI, 15 µg), and erythromycin (ERI, 15 µg).

The results for all antimicrobials, except ENR, ERI, CLI, and AMI, were interpreted according to CLSI M100™ guidelines [22]. ENR was interpreted according to CLSI VET01S™ guidelines [23], whereas AMI, ERI, and CLI were interpreted according to BrCAST criteria [24]. The minimum inhibitory concentration (MIC) for oxacillin was determined for CFO-resistant *Staphylococcus* species according to CLSI M100™ guidelines [22]. Accordingly, isolates of Enterobacterales and *S. aureus* were classified as MDR if they exhibited non-susceptibility (intermediate or resistant) to at least one agent in three or more antimicrobial classes [25]. Because no specific MDR definition is provided for non-*aureus Staphylococcus* species, the same criteria established for *S. aureus* were applied to these isolates.

### 2.4. Detection of Antimicrobial Resistance and Virulence Genes

Conventional polymerase chain reaction (PCR) assays were performed to detect the genes *mecA* and *blaZ* in staphylococcal isolates (*S. aureus*, *S. epidermidis*, and *S. ureilyticus*). Regarding virulence determinants, the genes *sea*, *seb*, *sec*, and *see* were investigated in *S. aureus* isolates to assess their potential for enterotoxin production associated with foodborne illness. In addition, the genes *eae* and *stx1*/*stx2* were screened in *E. coli* isolates to determine their classification as enteropathogenic *E. coli* (EPEC) or Shiga toxin-producing *E. coli* (STEC) pathotypes, respectively.

#### 2.4.1. DNA Extraction

Genomic DNA was extracted using a modified protocol based on Bag et al. [26]. For each isolate, 1.8 mL of an overnight bacterial culture was harvested by centrifugation at 6800× *g* for 5 min at 12 °C, and the resulting pellet was retained after discarding the supernatant. Cell lysis was initiated by resuspending the pellet in 700 µL of extraction buffer composed of 160 mM Tris-HCl (pH 8.0), 60 mM EDTA (pH 8.0), 20 mM NaCl, and 0.5% (*w*/*v*) SDS. Efficient disruption of bacterial cell walls was achieved through the combined use of lysozyme, mutanolysin, and lysostaphin, enabling lysis of both Gram-negative and Gram-positive bacteria. The suspensions were incubated at 65 °C for 40 min to promote complete lysis.

Following incubation, 300 µL of 5 M potassium acetate was added, and the mixtures were homogenized and kept on ice for 30 min to facilitate protein precipitation. Phase separation was then performed by adding 600 µL of chloroform:isoamyl alcohol (24:1, *v*/*v*), followed by centrifugation at 15,300× *g* for 10 min at 10 °C. The aqueous phase was carefully transferred to new tubes, and DNA was precipitated by adding 1000 µL of cold absolute ethanol, followed by gentle mixing and incubation at −20 °C for 12 h. DNA was recovered by centrifugation at 15,300× *g* for 17 min at 10 °C. The resulting pellet was washed with 70% (*v*/*v*) ethanol, air-dried at 55 °C, and finally resuspended in 30–50 µL of TE buffer (10 mM Tris-HCl, 1 mM EDTA; pH 8.0). DNA yield and purity were assessed using a NanoDrop One spectrophotometer (Thermo Scientific, Waltham, MA, USA).

#### 2.4.2. PCR Assays

PCR amplification was carried out following the protocol described by Towner et al. [27], with slight modifications. Reactions were prepared in a final volume of 25 µL containing 2.5 pmol of each primer, 0.2 mM of each dNTP, 1.5 mM MgCl_2_, 0.6 U of Taq DNA polymerase, and 4.0 µL of template DNA. Following amplification, PCR products were mixed with 5 µL of loading buffer (0.25% bromophenol blue in 50% glycerol) and resolved by electrophoresis on a 1.5% agarose gel supplemented with ethidium bromide (1 µg/mL) in Tris–Borate–EDTA (TBE) buffer. A 100 bp DNA ladder was included as a molecular size standard, and electrophoresis was conducted at 70 V for 60 min.

The target genes analyzed, the respective primer sequences, the strains used as positive controls, and the expected amplicon sizes are presented in Table 1. Amplification of the target genes was performed based on the protocols described in the references listed in Table 1, with minor modifications for *stx1*, *stx2*, and *eae*, for which a final extension step at 72 °C for 7 min was added. For *blaZ*, the protocol involved an initial denaturation step at 95 °C for 3 min, followed by 30 cycles of denaturation (94 °C for 1 min), annealing (55 °C for 1 min), and extension (72 °C for 1 min), with a final extension step at 72 °C for 7 min.

## 3. Results

### 3.1. Antimicrobial-Resistant Enterobacterales Isolated from Insects

**Figure 1:**
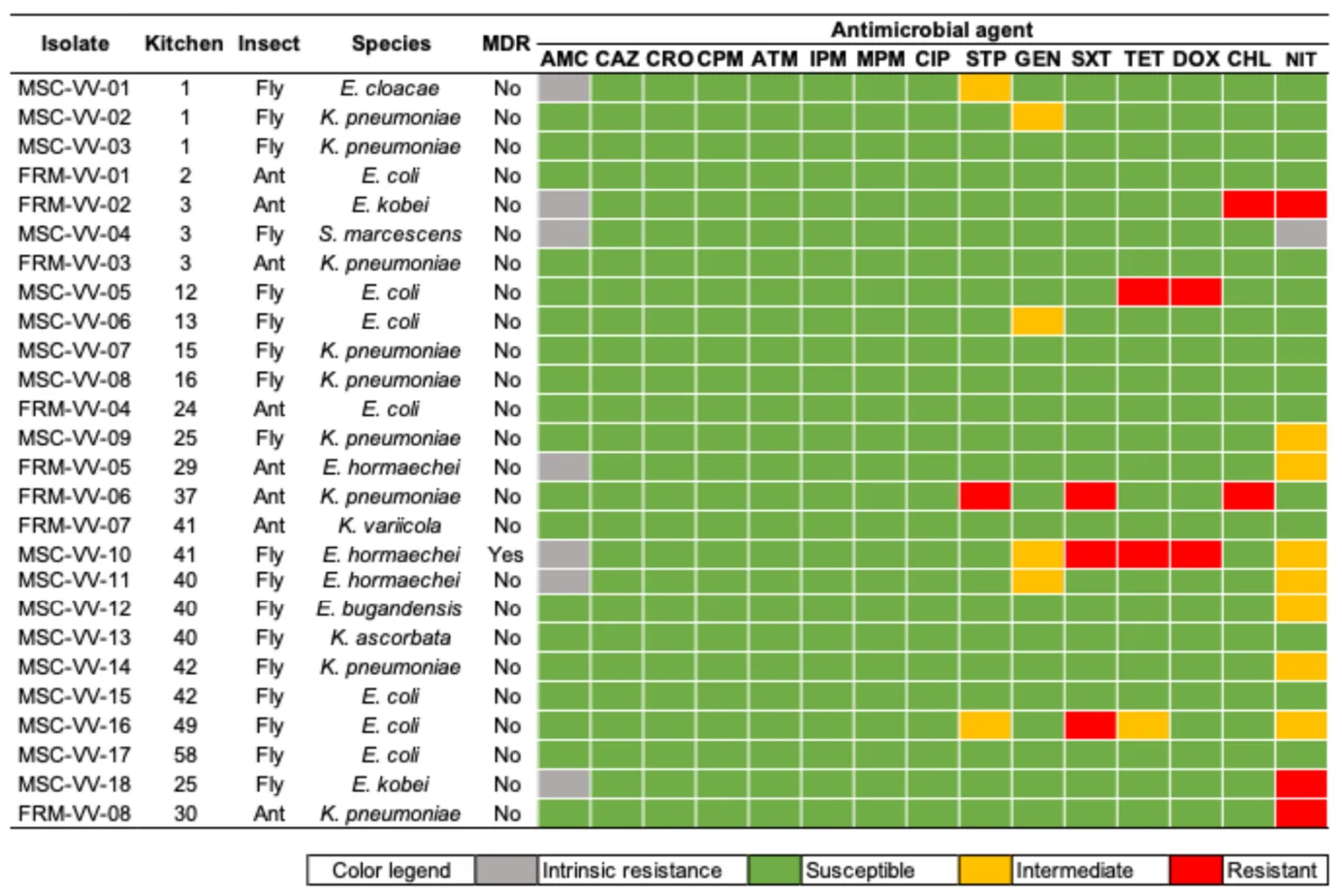
Identification results and AMR profiles of Enterobacterales isolated from flies and ants in domestic kitchens. MDR: multidrug resistant; AMC: amoxicillin–clavulanic acid; ATM: aztreonam; CAZ: ceftazidime; CIP: ciprofloxacin; CHL: chloramphenicol; CPM: cefepime; CRO: ceftriaxone; DOX: doxycycline; STP: streptomycin; GEN: gentamicin; IPM: imipenem; MPM: meropenem; NIT: nitrofurantoin; SXT: trimethoprim–sulfamethoxazole; and TET: tetracycline.

Twenty-six Enterobacterales isolates were recovered from 17 of the 58 domestic kitchens studied. These isolates originated from 18 flies and 8 ants. In two kitchens (named 3 and 41), Enterobacterales were isolated from both insect groups. Additionally, in four kitchens (named 1, 25, 40, and 42), isolates were recovered from more than one fly specimen. Overall, nine species belonging to the order Enterobacterales were identified. *Enterobacter bugandensis* (n = 1), *Enterobacter cloacae* (n = 1), *Kluyvera ascorbata* (n = 1), and *Serratia marcescens* (n = 1) were exclusively isolated from flies, whereas *Klebsiella variicola* (n = 1) was recovered only from ants. In contrast, *Enterobacter hormaechei* (n = 3), *Enterobacter kobei* (n = 2), *Escherichia coli* (n = 7), and *Klebsiella pneumoniae* (n = 9) were isolated from both flies and ants. Among these isolates, the *E. hormaechei* MSC-VV-10 was isolated from a fly and classified as MDR. Additionally, the non-MDR profiles, including resistance to aminoglycosides, tetracyclines, phenicols, and nitrofurans, were observed in flies and ants (Figure 1). No *E. coli* isolates carried the virulence genes *stx1*, *stx2*, and *eae*.

### 3.2. Diversity of Antimicrobial-Resistant Staphylococcus Species Recovered from Insects

A total of 45 staphylococci isolates were recovered from 25 of the 58 domestic kitchens studied, originating from 36 flies and 9 ants. In four kitchens (named 2, 13, 37, and 40), staphylococci were isolated from both insect groups. Additionally, in two kitchens (named 2 and 40), isolates were recovered from more than one ant specimen, whereas in 10 kitchens (named 3, 32, 36, 37, 38, 40, 41, 42, 50, and 56), isolates were obtained from different fly specimens. Overall, nine *Staphylococcus* species were identified. *S. aureus* (n = 5), *S. nepalensis* (n = 1), and *S. warneri* (n = 2) were exclusively isolated from flies, whereas *S. ureilyticus* (n = 1) was recovered only from ants. In contrast, *S. epidermidis* (n = 4), *S. gallinarum* (n = 5), *S. sciuri* (n = 11), *S. simulans* (n = 4), and *S. xylosus* (n = 12) were isolated from both flies and ants.

**Figure 2:**
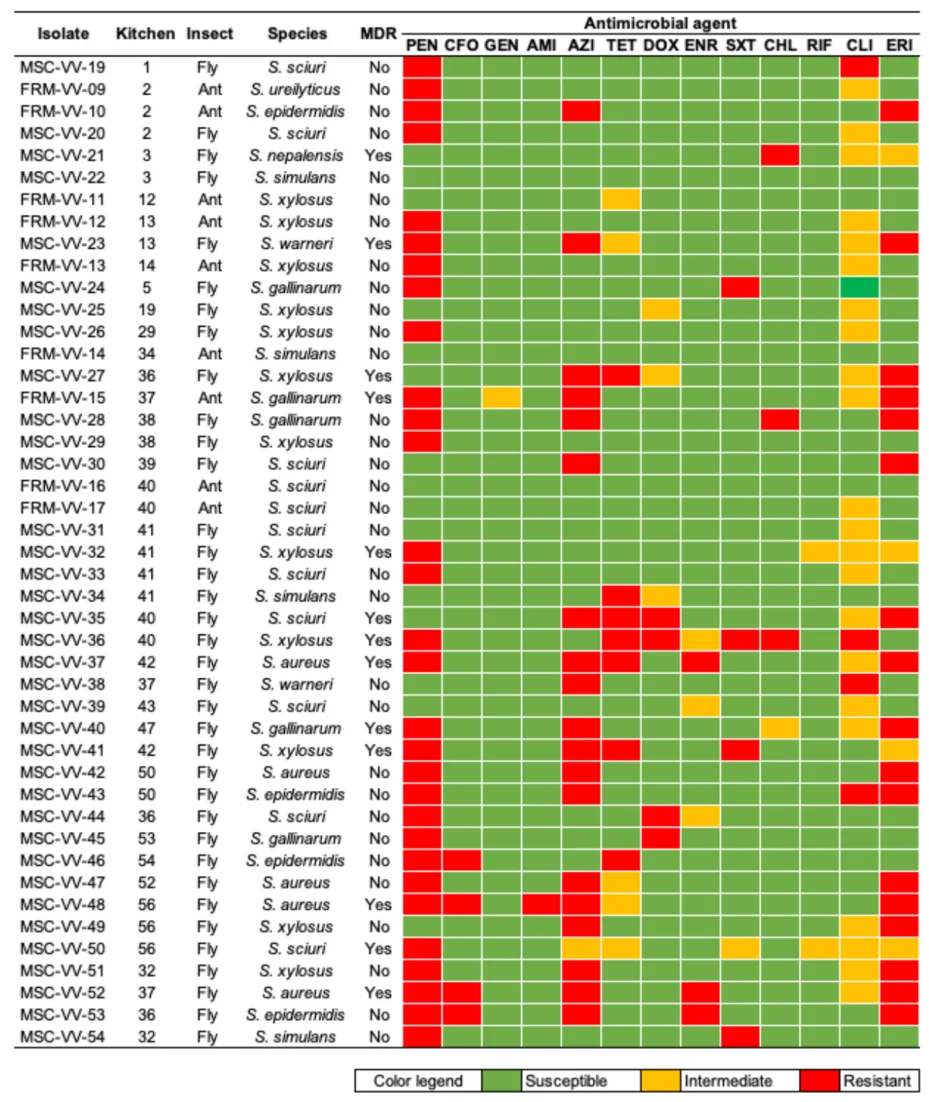
Identification results and AMR profiles of staphylococci isolated from flies and ants in domestic kitchens. MDR: multidrug resistant; AMI: amikacin; AZI: azithromycin; CFO: cefoxitin; CLI: clindamycin; CHL: chloramphenicol; DOX: doxycycline; ENR: enrofloxacin; ERI: erythromycin; GEN: gentamicin; PEN: penicillin; RIF: rifampicin; SXT: trimethoprim–sulfamethoxazole; and TET: tetracycline.

A total of 13 MDR staphylococci isolates were identified in this study. High rates of resistance to penicillin, azithromycin, and clindamycin were detected. Resistance to other antimicrobials was also observed. The MDR staphylococci comprised *S. aureus* (n = 3), *S. nepalensis* (n = 1), *S. sciuri* (n = 2), *S. warneri* (n = 1), *S. gallinarum* (n = 2), and *S. xylosus* (n = 4) isolated from flies and ants (Figure 2). Two *S. aureus* isolates (MSC-VV-48 and MSC-VV-52) and two *S. epidermidis* isolates (MSC-VV-46 and MSC-VV-53) were resistant to CFO. Accordingly, the MIC for oxacillin was determined, yielding values ranging from 1 to >256 mg/L. The *mecA* gene was investigated in these isolates, but it was not detected. The *blaZ* gene was detected in isolates MSC-VV-37, MSC-VV-42, MSC-VV-47, MSC-VV-48, MSC-VV-52, and MSC-VV-53, corroborating the observed penicillin resistance phenotype. No *S. aureus* isolates presented the virulence genes *sea*, *seb*, *sec*, and *see*.

## 4. Discussion

The isolation of 18 distinct bacterial species, comprising nine Enterobacterales and nine staphylococcal species, highlights the remarkable bacterial diversity carried by flies and ants in domestic kitchens. Several of the bacterial species identified in the present study, especially *K. pneumoniae*, *E. hormaechei*, *S. marcescens*, and *S. aureus*, are recognized as important opportunistic pathogens [32], with several also representing significant foodborne hazards [33,34,35]. These findings also support previous evidence indicating that insects may contribute to the contamination of food and food-contact surfaces through their frequent movement between contaminated substrates and food preparation areas [20]. The high frequency and diversity of staphylococci observed in this study are expected given that these bacteria are common colonizers of human and animal skin and are frequently recovered from indoor environments. Their persistence on environmental surfaces may facilitate acquisition and dissemination by crawling and flying insects.

House flies have coprophagic and saprophagous feeding habits and frequently visit feces, decomposing organic matter, garbage, sewage, and food residues, allowing them to acquire bacteria from multiple contaminated substrates before landing on food or food-contact surfaces. Their body surface, including the legs, wings, mouthparts, and body setae, can mechanically carry microorganisms, while their feeding behavior further enhances bacterial dissemination. During feeding, flies regurgitate digestive fluids and may defecate on food and surrounding surfaces, promoting the deposition and potential transfer of viable bacteria [16,20]. Female flies may contribute disproportionately to bacterial dissemination because they frequently visit highly contaminated oviposition sites and, owing to their ability to travel distances of up to 20 km during their lifetime, can spread microorganisms over wide areas [36,37]. Consequently, flies may disseminate a wide variety of bacterial species, including opportunistic and foodborne pathogens, as well as antimicrobial-resistant strains, thereby representing an important route for the environmental spread of clinically important ARBs.

Ants have also been recognized as potential carriers of microorganisms of public health importance. Their small body size enables them to access cracks, crevices, food storage areas, and food-contact surfaces, whereas their highly mobile and social foraging behavior promotes frequent movement between contaminated substrates and food preparation environments. During foraging, ants may acquire microorganisms on their exoskeleton or through contact with contaminated materials and subsequently transfer them to foods, utensils, and food-contact surfaces. Consequently, these insects have been associated with the dissemination of a wide range of bacteria, including foodborne pathogens and opportunistic species, highlighting their potential role in microbial cross-contamination in domestic and food-handling environments [15].

Previous studies have demonstrated that flies may serve as carriers of ARBs in a variety of environments, including slaughterhouses and meat-processing facilities [16], hospitals [17], areas surrounding healthcare facilities [18], livestock farms [19], and kitchens [14], whereas evidence regarding ants has remained largely restricted to hospital settings [21]. Building upon these observations, the present study demonstrates that flies and ants collected from domestic kitchens can carry MDR bacteria. To the best of our knowledge, this is the first study reporting MDR bacteria simultaneously recovered from flies and ants collected in domestic kitchens. Accordingly, these results show the potential role of household insects as reservoirs and possible disseminators of opportunistic bacterial pathogens and AMR.

The identification of clinically important bacteria and multidrug resistance in household insects reinforces the potential role of household insects in transporting bacteria commonly associated with healthcare settings into domestic environments and highlights the importance of implementing effective food hygiene practices in domestic kitchens. Proper food storage, regular cleaning and disinfection of food-contact surfaces and utensils, prevention of cross-contamination, and adequate vector control are essential measures to reduce the transmission of foodborne microorganisms. These practices are consistent with Good Manufacturing Practices principles and represent critical preventive strategies to improve food safety in household environments, which remain an important setting for foodborne disease transmission [10,38,39,40].

Insect-derived *S. aureus* and *S. epidermidis* isolates were classified as methicillin-resistant based on cefoxitin susceptibility testing, corresponding to MRSA and MRSE phenotypes. The main related AMR mechanism, mediated by the *mecA* gene, was not found, suggesting the presence of non-investigated mechanisms, including the possible involvement of *mecC*, which was not investigated in the present study, or mutations in native penicillin-binding proteins [41]. Environmental staphylococci remain considerably less investigated than clinical isolates, and therefore, the genetic basis underlying β-lactam resistance in these bacteria is still incompletely understood. Therefore, the molecular characterization of additional determinants associated with MRSA and MRSE should be considered in future studies.

All five *S. aureus* isolates were resistant to penicillin and carried the *blaZ* gene. Among the four *S. epidermidis* isolates, all were resistant to penicillin; however, only one isolate was positive for *blaZ*. Other *Staphylococcus* species were also resistant to penicillin and did not harbor the *blaZ* gene. The complete agreement between the penicillin resistance phenotype and the presence of *blaZ* among *S. aureus* isolates is consistent with previous studies showing that *blaZ* is the major determinant responsible for penicillin resistance in this species [42]. Regarding coagulase-negative staphylococci (CoNS), previous studies have demonstrated that not all penicillin-resistant *S. epidermidis* isolates harbor the *blaZ* gene [43,44], suggesting that additional mechanisms may contribute to the penicillin-resistant phenotype in this species. Likewise, the penicillin-resistant but *blaZ*-negative *S. ureilyticus* isolate indicates that β-lactam resistance cannot always be explained solely by the presence of this gene. These findings highlight the molecular diversity of β-lactam resistance among environmental staphylococci and reinforce the need for broader genomic investigations to better characterize resistance determinants circulating in CoNS species.

This study has some limitations that should be considered when interpreting the findings. Although a relatively large number of households was included, sampling was restricted to a single geographical region, which may limit the generalizability of the results. In addition, taxonomic identification of the collected ants and flies was not performed to minimize sample manipulation and avoid potential contamination during processing. Molecular analyses were limited to selected AMR and virulence determinants and to the most clinically important bacterial species, while whole-genome sequencing was not performed, precluding comprehensive characterization. Future studies should combine culture-dependent approaches with genomic analyses to provide a more comprehensive assessment of the bacterial communities and AMR profiles associated with household insects. Furthermore, integrated sampling of insects, food-contact surfaces, utensils, and food items would help clarify possible transmission pathways and elucidate the role of household insects in the dissemination of ARBs within domestic kitchen environments.

## 5. Conclusions

This study demonstrates that flies and ants inhabiting domestic kitchens can carry a diverse bacterial community, including clinically important pathogens and MDR bacteria. These findings provide further evidence that household insects may act as mechanical vectors of ARBs, facilitating their dissemination between contaminated substrates, food-contact surfaces, utensils, and food within domestic environments. By identifying MDR bacteria in insects collected from household kitchens, this study expands current knowledge on the ecology of antimicrobial resistance and reinforces the importance of considering domestic environments within the One Health context. Although the epidemiological significance of this transmission route remains to be fully elucidated, our findings highlight the need for effective household hygiene practices, appropriate insect control, and increased surveillance in community settings to better understand the spread of ARBs along the food chain.

## Figures and Tables

**Table 1:** Primer sequences, amplicon sizes, and positive controls for AMR and virulence determinants.

Gene	Primer Sequence (5′-3′) ^1^	Amplicon Size (bp)	Positive Control	Reference
*E. coli*				
*stx1*	F: AGAGCGATGTTACGGTTTG	388	Clinical isolate (bovine)	[28]
	R: TTGCCCCCAGAGTGGATG			
*stx2*	F: TGGGTTTTTCTTCGGTATC	807	ATCC^®^ 43895	[28]
	R: GACATTCTGGTTGACTCTCTT			
*eae*	F: AGGCTTCGTCACAGTTG	570	ATCC^®^ 43895	[28]
	R: CCATCGTCACCAGAGGA			
*Staphylococcus* spp.				
*mecA*	F: AAAATCGATGGTAAAGGTTGGC	532	ATCC^®^ 43300	[29]
	R: AGTTCTGCAGTACCGGATTTGC			
*blaZ*	F: ACTTCAACACCTGCTGCTTTC	173	ATCC^®^ 33592	[30]
	R: TGACCACTTTTATCAGCAACC			
*sea*	F: CCTTTGGAAACGGTTAAAACG	127	ATCC^®^ 13565	[31]
	R: TCTGAACCTTCCCATCAAAAAC			
*seb*	F: TCGCATCAAACTGACAAACG	477	ATCC^®^ 14458	[31]
	R: GCAGGTACTCTATAAGTGCCTGC			
*sec*	F: CTCAAGAACTAGACATAAAAGCTAGG	271	ATCC^®^ 19095	[31]
	R: TCAAATCGGATTACCATTATCC			
*see*	F: TAGATAAAGTTAAAACAAGC	178	ATCC^®^ 27664	[31]
	R: TAACTTACCGTGGACCCTTC			

^1^ F, forward; R, reverse.

## Data Availability

The datasets generated and/or analyzed during the current study are available from the corresponding authors on reasonable request.

## Supplementary Materials

The following supporting information can be downloaded at: https://www.mdpi.com/article/10.3390/microorganisms14091928/s1. Supplementary Table S1: Number of domestic kitchens and flies and ants captured in each one.
